# Elevated Circulating Trimethylamine N-Oxide Levels Contribute to Endothelial Dysfunction in Aged Rats through Vascular Inflammation and Oxidative Stress

**DOI:** 10.3389/fphys.2017.00350

**Published:** 2017-05-30

**Authors:** Tiejun Li, Yanli Chen, Chaojun Gua, Xiaodong Li

**Affiliations:** Department of Cardiology, Shengjing Hospital of China Medical UniversityShenyang, China

**Keywords:** aging, trimethylamine N-oxide, endothelial dysfunction, inflammation, oxidative stress

## Abstract

Vascular endothelial dysfunction, a characteristic of the aging process, is an important risk factor for cardiovascular disease in aging. Although, vascular inflammation and oxidative stress are major contributors to endothelial dysfunction in aging, the underlying mechanisms during the aging process are not fully understood. Accumulating evidence reveals that gut microbiota-dependent metabolite trimethylamine-N-oxide (TMAO) is implicated in the pathogenesis of many cardiovascular diseases. We tested the hypothesis that aging increases circulating TMAO levels, which induce vascular inflammation and oxidative stress, resulting in age-associated endothelial dysfunction. Old (22-mo-old) and young (4-mo-old) Fischer-344 rats were treated without (control) or with 1.0% 3,3-Dimethyl-1-butanol (DMB, an inhibitor of trimethylamine formation) in drinking water for 8 weeks. Compared with young control group, old control group had markedly higher plasma TMAO levels, which were reduced by DMB treatment. Endothelium-dependent relaxation of aorta in response to acetylcholine was impaired in old control group compared with young control group as indicated by decreased maximal relaxation (E_max_) and reduced area under the curve (AUC). E_max_ and AUC were both normalized in old rats treated with DMB. No difference in endothelial-independent relaxation in response to sodium nitroprusside was observed among groups. Molecular studies revealed that old control group exhibits increased expression of proinflammatory cytokines and superoxide production, and decreased expression of endothelial nitric-oxide synthase (eNOS) in the aorta, all of which were restored by DMB treatment. These results suggest that aging increases circulating TMAO levels, which may impair eNOS-derived NO bioavailability by increasing vascular inflammation and oxidative stress, contributing to aging-associated endothelial dysfunction.

## Introduction

Aging is an independent risk factor for cardiovascular disease (Lakatta and Levy, 2003; Herrera et al., 2010; El Assar et al., 2012; Donato et al., 2015), which is the leading cause of death worldwide. Advancing age is associated with complex alterations in vascular structure and function (Yildiz, 2007; Herrera et al., 2010; Donato et al., 2015), and the progressive decline of endothelial function with advanced age has been demonstrated to play a major role in the development of cardiovascular disease (Herrera et al., 2010; Donato et al., 2015). The aging process results in an imbalance between vasodilating and vasoconstricting substances that are produced by the endothelium (Herrera et al., 2010; Donato et al., 2015), and this disruption in balance is largely due to the reduced bioavailability of nitric oxide (NO), which is produced mainly from L-arginine by endothelial nitric-oxide synthase (eNOS) and is one of the most important vasodilatory and anti-atherosclerotic molecules (Herrera et al., 2010; Seals et al., 2011; Donato et al., 2015).

Advancing age is accompanied by increased vascular inflammation and oxidative stress, which have been suggested to be the major contributes to aging-associated reduction in NO bioavailability and endothelial dysfunction (Herrera et al., 2010; Donato et al., 2015). However, the mechanisms underlying vascular inflammation and oxidative stress with advancing age are not fully understood.

Accumulating evidence reveals that gut microbiota-dependent metabolite trimethylamine-N-oxide (TMAO) is implicated in the pathogenesis of many cardiovascular diseases (Tang and Hazen, 2014, 2017; Organ et al., 2016; Senthong et al., 2016). For example, elevated TMAO levels are strongly correlated with increased risk of adverse cardiovascular outcomes, including heart attack, stroke and death risk (Tang and Hazen, 2014, 2017; Tang et al., 2015; Kitai et al., 2016; Li et al., 2017). Elevated TMAO levels also exacerbate pressure overload-induced heart failure (Organ et al., 2016) and prolong hypertensive effect of angiotensin II (Ufnal et al., 2014). Moreover, elevated TMAO levels promote vascular inflammation and oxidative stress (Seldin et al., 2016; Sun et al., 2016), impair vascular function (Hu et al., 2015; Sun et al., 2016) and induce atherosclerosis (Wang et al., 2011, 2015; Chen et al., 2016). To date, however, no studies have examined the role of TMAO in aging-associated cardiovascular changes. In the present study, we examined whether circulating TMAO levels are elevated with aging and, if so, whether elevated TMAO levels induce vascular inflammation and oxidative stress, resulting in age-associated endothelial dysfunction.

## Methods

### Animals

Male Fisher-344 rats were purchased from Vital River (A Charles River Company, Beijing, China). All animals were individually housed under 12-h light–12-h dark conditions with food and water available *ad libitum*. All experimental protocols were approved by the Institutional Animal Care and Use Committee of China Medical University, and were conducted in accordance with the “Guiding Principles for Research Involving Animals and Human Beings.”

### Protocol

Thirty-six young (~4-mo-old) and 36 old (~22-mo-old) animals were randomly divided into four experimental groups as follows (*n* = 18 for each group): (1) young control rats (Y-CON), (2) young rats treated with 3,3-Dimethyl-1-butanol (DMB, an inhibitor of trimethylamine formation) (Y-DMB), (3) old control rats (O-CON), and (4) old rats treated with DMB (O-DMB). 1.0% DMB was given in drinking water for 8 weeks and this dose of DMB has been demonstrated to effectively inhibit trimethylamine formation and reduce circulating TMAO levels in rodent (Wang et al., 2015).

At the end of the study protocol, the animals were weighed and then sacrificed by decapitation under deep anesthesia. Blood samples were collected for TMAO measurement. The thoracic aorta was quickly dissected from animal and placed in ice-cold physiological saline solution (PSS). After cleaning of fat and connective tissues, thoracic aorta was cut into 3-mm ring sections for assessment of vascular function. Some ring sections of aorta were denuded by gently rubbing the luminal surface with forceps to further verify the role endothelium in mediating ACh-induced relaxation or to assess vascular smooth muscle function. The remainder of the aortic tissue was used for detection of superoxide production or snap-frozen in liquid nitrogen and stored at −80°C for molecular study.

### Measurement of plasma TMAO

Plasma levels of TMAO were measured using liquid chromatography coupled with triple-quadrupole mass spectrometry as described previously (Ufnal et al., 2014). Briefly, 10 μL of plasma was transferred to a 1.5 mL silanized conical test tube followed by addition of 1,000 μL of acetone with trimethylamine -13C3,15N Hydrochloride (300 ng/ml) to precipitate protein and extract analytes. After centrifugation of the mixture at 18626 RCF for 2 min, the supernatant was transferred to the vial and analyzed by LC/MS/MS. Some samples that exceeded concentration of the highest calibration point were diluted by adding 2.5 μL of plasma, 7.5 μL of Mili-Q water and 1,000 μL of acetone with trimethylamine -13C3,15N Hydrochloride and the results were corrected accordingly.

### Assessment of vascular function

Aortic rings were suspended on a wire myograph (model 610 M; Danish Myotechnology) for isometric tension measurement, as described previously (Martens et al., 2014; Guers et al., 2017). Each ring was continuously oxygenated with a gas mixture of 95% O_2_ and 5% CO_2_ and kept under physiological conditions at 37°C and pH 7.4 in PSS. The ring was stretched to an optimal baseline tension of 30 mN and then allowed to equilibrate for 60 min. Following equilibration, ring was constricted by phenylephrine (3 × 10^−7^ M) and then relaxed by ACh (10^−4^ M) to test the viability of the endothelium. After the viability of the endothelium was confirmed, each ring was again constricted by phenylephrine (3 × 10^−7^ M) and then relaxed by cumulative additions of ACh (10^−9^ to 10^−5^ M). Following the completion of the first dose-response experiment, ring was washed with PSS every 10 min for 30 min and then treated with 10 μM of a non-selective NOS inhibitor NG-nitro-L-arginine methyl ester (L-NAME, Sigma-Aldrich, St. Louis, MO, USA) to test whether impaired endothelium-dependent vasodilation was mediated by NO. Ring was again constricted and dose dependently relaxed by ACh as mentioned above. Finally, the relaxant responses to ACh (10^−9^ to 10^−5^ M) or sodium nitroprusside (SNP, 10^−9^ to 10^−5^ M) were performed in the endothelium-denuded rings pre-constricted by phenylephrine (3 × 10^−7^ M) to further verify that impaired vasodilation in aging was mediated by endothelium or to examine endothelium-independent vasodilation.

### Western blot analysis

Protein levels of pro-inflammatory cytokines tumor necrosis factor (TNF)-α and interleukin (IL)-1β and protein levels of eNOS and phosphorylated eNOS (Ser1177) in the aortic tissue were analyzed by western blot. Briefly, the aortic tissue was homogenized in a mammalian tissue lysis buffer with protease inhibitor (Sigma-Aldrich, St. Louis, MO, USA). The protein concentration was assessed by the Bradford method. Samples were loaded in a 12% SDS-polyacrylamide gels and then transferred to a polyvinylidene difluoride membranes (Millipore Corporation, Bedford, MA, USA). After blocking for 1 h in 5% non-fat dry milk, the membranes were immunoblotted with primary antibodies to TNF-α, IL-1β (Cell Signaling Technology, Beverly, MA, USA), eNOS (BD Biosciences, San Jose, CA, USA), phosphorylated eNOS Ser1177 (BD Biosciences, San Jose, CA, USA) and β-actin (Santa Cruz Biotechnology Inc., Santa Cruz, CA, USA) overnight at 4°C. The membranes were washed and then incubated with HRP-conjugated secondary antibody (Santa Cruz Biotechnology Inc., Santa Cruz, CA, USA) at room temperature for 1 h. Membranes were incubated with the enhanced chemiluminescence detection reagents (Amersham, Arlington Heights, IL, USA) and band densities were analyzed with ImageJ software (National Institutes of Health, Bethesda, Maryland, USA). All data were expressed as relative units normalized to β-actin.

### Detection of superoxide production

Dihydroethidium (DHE), an oxidative fluorescent dye, was used to detect superoxide production in the aorta by fluorescence microscope as described previously (Hezel et al., 2016). Briefly, the aorta was immediately frozen at −80°C for 1 h, blocked in the coronal plane, and sectioned into 10-μm slices with a cryostat. The sections were mounted on microscope slides and incubated with DHE (2 μmol/L, Molecular Probes) for 30 min at 37°C in a light-protected humidified chamber. The sections were examined by a Zeiss fluorescence microscope and images were acquired at 40 × magnification. For each aortic ring, DHE fluorescence was calculated from four separate fields from each quadrant of the ring and quantified using image J software (NIH, Bethesda, MD, USA).

### Statistical analysis

All data are presented as means ± SEM. Statistical analysis was performed using a two-way ANOVA followed by Tukey's *post-hoc* test. Dose-response curves were created for vascular function data with GraphPad Prism 7 (GraphPad Software, La Jolla, CA, USA) and normalized to percent relaxation, as described previously (Martens et al., 2014; Guers et al., 2017). Dose-response curves were fit with a non-linear regression line, and maximal relaxation (E_max_), LogEC_50_ as the negative logarithm of the drug that produced 50% of E_max_ and the area under the curve (AUC) were calculated. Differences were considered significant if *P* < 0.05.

## Results

### Effects of aging and DMB on body weight

At the end of the experimental protocol, body weight was significantly higher in both groups of old rats than the young groups (Y-CON, 358 ± 25 g; Y-DMB, 350 ± 30 g; O-CON, 438 ± 21 g; O-DMB, 433± 29 g; *P* < 0.05). DMB treatment had no effect on body weight, since body weight did not differ between the DMB groups and their respective control groups.

### Effects of aging and DMB on plasma TMAO levels

**Figure 1:**
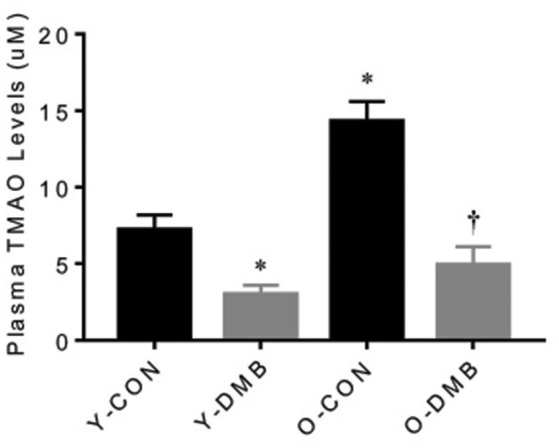
Effects of aging and 3,3-Dimethyl-1-butanol (DMB, an inhibitor of trimethylamine formation) on circulating TMAO Levels. Old rats had higher circulating TMAO levels than young rats. DMB treatment reduced circulating TMAO levels in both young and old rats. Data are expressed as mean ± SE (*n* = 9–10 for each group). ^*^P < 0.05 vs. Y-CON; ^†^*P* < 0.05, O+DMB vs. O-CON.

Compared with young control group, old control group had markedly higher levels of plasma TMAO (Y-CON, 6.41 ± 1.27 μM vs. O-CON, 14.30 ± 1.52 μM; *P* < 0.05; Figure 1). Compared with respective control groups, DMB treatment significantly (*P* < 0.05) reduced plasma TMAO levels not only in old animals (O-DMB, 5.97 ± 1.31 μM) but also in young animals (Y-DMB, 3.85 ± 0.73 μM).

### Effects of aging and DMB on vascular function

**Figure 2:**
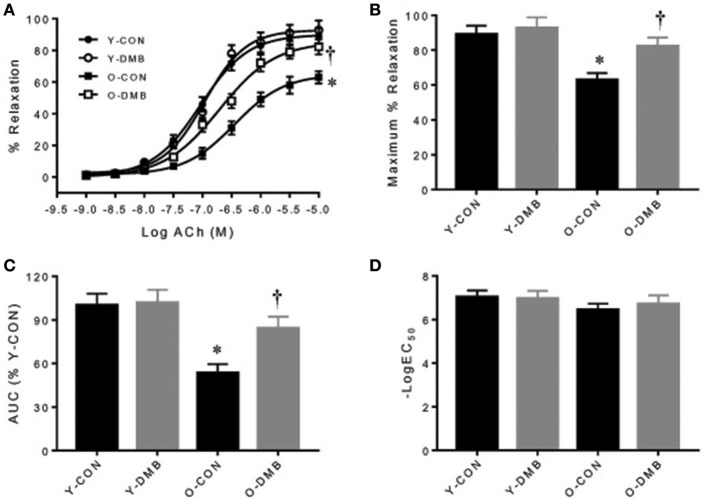
Effects of aging and DMB on endothelial-dependent relaxation. **(A)** Dose responses of endothelium-intact aortic rings to acetylcholine (ACh); **(B)** Maximal relaxation (E_max_) to ACh; **(C)** Area under the curve (AUC), and **(D)** Half maximal effective concentration (LogEC_50_). Data are expressed as mean ± SE (*n* = 9–10 for each group). ^*^P < 0.05 vs. Y-CON; ^†^*P* < 0.05, O+DMB vs. O-CON.

**Figure 3:**
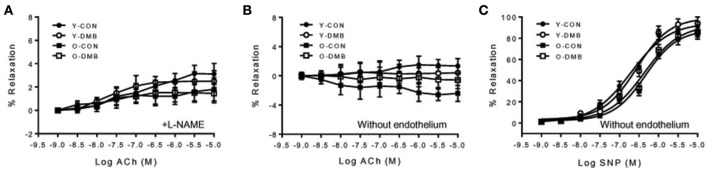
**(A)** Effects of aging and DMB on endothelial-dependent relaxation to ACh in endothelium-intact aortic rings in the presence of a non-selective nitric oxide synthase (NOS) inhibitor NG-nitro-L-arginine methyl ester (L-NAME); **(B)** effects of aging and DMB on endothelial-dependent relaxation to ACh in endothelium-denuded aortic rings, and **(C)** effects of aging and DMB on endothelial-independent relaxation to sodium nitroprusside (SNP) in endothelium-denuded aortic rings. Data are expressed as mean ± SE (*n* = 6–10 for each group).

The dose-response curves of ACh-induced endothelium-dependent relaxation in intact aortic rings are presented in Figure 2A. Old control group showed a significant reduction in ACh-induced relaxation compared with all other groups. E_max_ was decreased by 30% in old control group, compared with young control group (O-CON, 62 ± 4 vs. Y-CON, 89 ± 5%; *P* < 0.05), but was elevated in old DMB group (Figure 2B), resulting in no difference (*P* > 0.05) between old DMB group (O-DMB: 83 ± 5%) and young control group or young DMB group (Y-DMB: 93 ± 7%). AUC was significantly reduced by 47% in the old control group compared with young control group (%Y-CON: O-CON, 53 ± 6 vs. Y-CON, 100 ± 8%; *P* < 0.05) but was rescued by DMB treatment (O-DMB: 84 ± 8%, *P* < 0.05 vs. O-CON; Figure 2C). There was no significant difference in logEC_50_ among four groups (Figure 2D). Notably, pre-treatment with L-NAME abolished ACh-induced relaxation in all groups (Figure 3A). When aortic rings were denuded to examine relaxation in the absence of the endothelium, ACh-induced relaxation was impaired for all groups, but no difference in relaxation was found among four groups (Figure 3B). There was no significant difference in basal tone or phenylephrine pre-constriction across four groups (data not shown).

The dose-response curves of sodium nitroprusside-induced endothelium-independent relaxation in endothelium-denuded aortic rings are shown in Figure 3C. There was no significant difference between the groups in response to sodium nitroprusside as analyzed by AUC (%Y-CON: Y-CON, 100 ± 0.07; Y-DMB, 101 ± 0.05; O-CON, 81 ± 0.07; OPTH, 84 ± 0.09%), E_max_ (YC, 95 ± 3; YPTH, 99 ± 2; OC, 89 ± 2; OPTH, 92 ± 3%), and logEC_50_ (YC, −6.7 ± 0.2; YPTH, −6.6 ± 0.1; OC, −6.3 ± 0.2; OPTH, −6.4 ± 0.2%), suggesting no age- or DMB treatment-related alteration in endothelium-independent relaxation.

### Effects of aging and DMB on expression of proinflammatory cytokines and eNOS in the aorta

**Figure 4:**
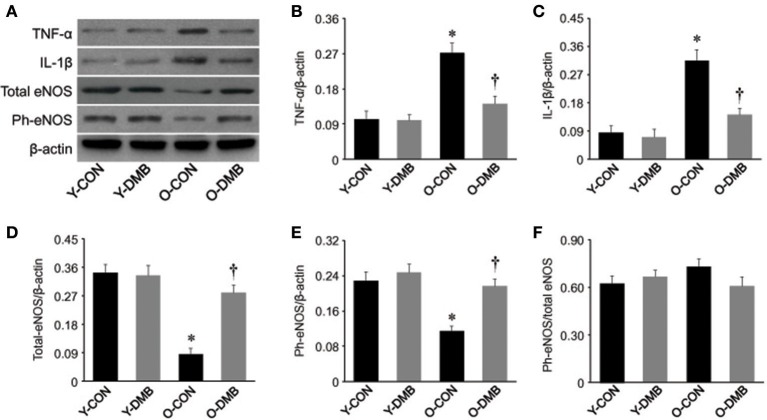
Effects of aging and DMB on expression of pro-inflammatory cytokines tumor necrosis factor (TNF)-α (B), interleukin (IL)-1β (C), total eNOS (D), phosphorylated (Ph-) eNOS (E), and the ratio of Ph-eNOS to total eNOS (F) in the aorta. Representative Western blots from each group are shown in **(A)**. Data are expressed as mean ± SE (*n* = 7 for each group). ^*^P < 0.05 vs. Y-CON; ^†^*P* < 0.05, O+DMB vs. O-CON.

Aging is associated with chronic inflammation, which may lead to impaired endothelial function by downregulating eNOS activity and has been considered as a major initial step in the development of atherosclerosis (Herrera et al., 2010; Donato et al., 2015). To examine whether elevated TMAO levels mediate vascular inflammation and eNOS activity in aging, we measured protein levels of two key proinflammatory cytokines TNF-α and IL-1β and activity of eNOS in the aorta. As shown in Figure 4, in the aorta of old control group, protein levels of TNF-α (Figure 4B) and IL-1β (Figure 4C) were significantly (*P* < 0.05) increased by 63 and 72%, respectively, while protein levels of total eNOS (Figure 4D) and phosphorylated eNOS (Figure 4E) were significantly (*P* < 0.05) decreased by 75 and 50%, respectively, compared with young control group. DMB treatment of old group reversed the protein levels of above parameters to the same extent as those observed in young control group. There were no significant differences (*P* > 0.05) in protein levels of TNF-α, IL-1β, total eNOS, and phosphorylated eNOS in the aorta between two young groups. In addition, when phosphorylated eNOS levels were corrected by total eNOS levels, there were no significant differences (*P* > 0.05) across four experimental groups (Figure 4F).

### Effects of aging and DMB on superoxide production in the aorta

**Figure 5:**
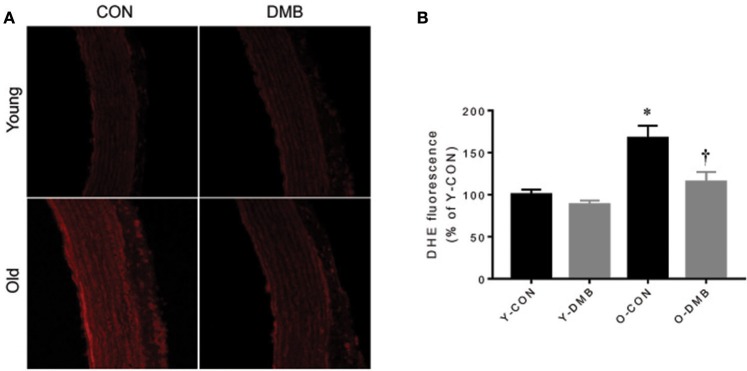
Effects of aging and DMB on superoxide production. NADPH oxidase-derived superoxide production in the aorta was detected by dihydroethidium (DHE). **(A)** Representative DHE images from each group; **(B)** Quantitative comparison of DHE fluorescence among groups. Data are expressed as mean ± SE (*n* = 7 for each group). ^*^P < 0.05 vs. Y-CON; ^†^*P* < 0.05, O+DMB vs. O-CON.

In addition to impairing eNOS activity, aging-associated inflammation may decrease NO bioavailability by increasing vascular superoxide production in the aorta (Herrera et al., 2010; Donato et al., 2015; Hezel et al., 2016). To determine whether elevated TMAO levels contribute to excessive superoxide production in aging, we next assessed intracellular superoxide production in the aorta using DHE. We observed a 40% higher intensity of DHE fluorescence in the aortic wall of old control animals when compared with young control animals (*P* < 0.05, Figure 5). Of note, aging-associated increase in DHE fluorescence intensity in the aortic wall of old animals was prevented by treatment with DMB, which had no effect in young animals.

## Discussion

The novel findings of this study are as follows: (1) compared young rats, old rats have higher plasma TMAO levels, which are prevented by treatment with DMB (an inhibitor of TMA formation); (2) endothelium-dependent but not endothelium-independent relaxation in the aorta is impaired in old rats, and is normalized by treatment with DMB; (4) old rats exhibit increased proinflammatory cytokines and superoxide production, and decreased eNOS expression in the aorta, all of which are restored by treatment with DMB. Taken together, these data suggest that circulating levels of gut microbiota-dependent metabolite TMAO are increased with aging, which may impair eNOS-derived NO bioavailability by increasing vascular inflammation and oxidative stress, resulting in aging-associated endothelial dysfunction.

Recent studies highlight the participation of gut microbiota in the pathogenesis of many diseases (Tang and Hazen, 2014; Adnan et al., 2017; Marques et al., 2017). The gut microbiota regulates multiple host physiological pathways, and most of effects are mediated through metabolites that act as energy sources, signaling molecules, receptor ligands and substrates for host enzymes (Jones, 2016; Anbazhagan et al., 2017). A growing body of evidence demonstrates that TMAO, a gut microbiota-generated phosphatidylcholine metabolite, is independently associated with the development of cardiovascular disease, including atherosclerosis and hypertension (Gregory et al., 2015; Wang et al., 2015; Senthong et al., 2016; Li et al., 2017). TMAO is derived from trimethylamine (TMA), which is produced by gut microbiota from dietary choline and phosphatidylcholine. After absorption in the gut, TMA reaches the liver where it is converted to TMAO by hepatic flavin-containing monooxygenases (FMOs; Wang et al., 2011; Koeth et al., 2013). Changes of gut microbiota composition, called dysbiosis, may lead to higher levels of TMAO (Kitai et al., 2016). Aging process has been shown to induce alterations in composition, diversity and functional features of gut microbiota, which are associated with aging-related decline in immune system functioning and chronic inflammation (Vaiserman et al., 2017). In the present study, we found that old control animals had significantly higher levels of plasma TMAO than young control animals, indicating that aging process elevates circulating TMAO levels. To our knowledge, this is the first study demonstrating that aging is associated with elevated circulating TMAO levels. We speculate that aging-induced dysbiosis may account for elevated circulating TMAO levels observed in this study.

Endothelial dysfunction has become a hallmark of vascular damage in advancing age and is a major risk factor for the development of atherosclerosis and hypertension (Herrera et al., 2010; Donato et al., 2015). Our results showed that ACh-induced endothelium-dependent relaxation was reduced in the aorta of older control animals compared with young control animals, whereas sodium nitroprusside-induced endothelium-independent relaxation was comparable between two groups. These results are consistent with the finding of previous studies (Martens et al., 2014; Guers et al., 2017), suggesting that endothelial dysfunction is an early and important mechanism that predisposes to atherothrombotic damage and thus contributes to the occurrence of cardiovascular events. Importantly, we found that impaired endothelium-dependent relaxation observed in old animals was prevented by treatment with DMB, which inhibited aging-induced increases in plasma TMAO levels. Notably, DMB treatment did not alter endothelium-independent relaxation in both old and young animals. These findings clearly demonstrate that aging-induced increases in circulating TMAO levels contribute to endothelial dysfunction during aging process.

**Figure 6:**
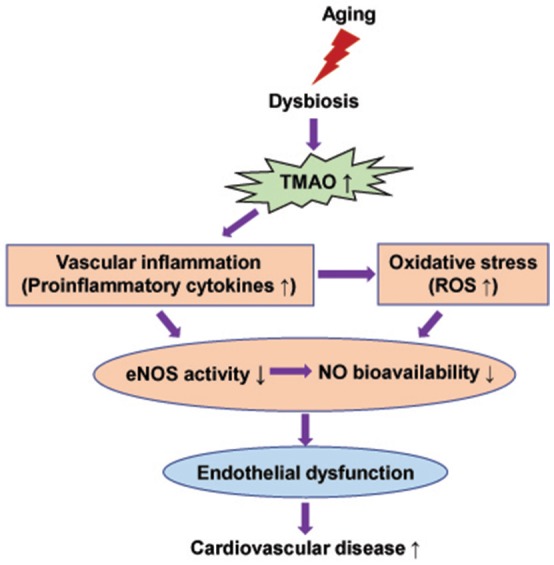
Schematic diagram showing possible mechanisms by which elevated TMAO levels induce endothelial dysfunction in aging.

Decreased NO bioavailability due to impaired eNOS-derived NO production or augmented oxidative stress has been suggested as central event, contributing to the age-associated endothelial dysfunction (Herrera et al., 2010; Cau et al., 2012; Silva et al., 2012; Donato et al., 2015). NO is produced in endothelial cells from L-arginine by the enzyme eNOS. Lower expression or activity of eNOS can decrease NO production (Tschudi et al., 1996; Barton et al., 1997; Csiszar et al., 2002; Soucy et al., 2006). In addition, increased superoxide anion (O2^−^), a ROS produced mainly by NADPH oxidase, can rapidly inactivate NO (Harrison, 1997; Price et al., 2000). Increased proinflammatory cytokines, a hallmark of aging, have been shown to downregulate eNOS activity in endothelial cells (Yoshizumi et al., 1993; Heeringa et al., 2002; Herrera et al., 2010) and cause vascular oxidative stress, leading to vascular damage in aging (Csiszar et al., 2007; Zhang et al., 2009; Herrera et al., 2010). Recent studies demonstrated that TMAO promotes vascular inflammation and oxidative stress, inhibits eNOS expression and activity and reduces NO production, which are associated with endothelial dysfunction and atherosclerosis (Wang et al., 2011; Hu et al., 2015; Chen et al., 2016; Seldin et al., 2016; Sun et al., 2016). Interventions to reduce circulating TMAO levels prevent the development of atherosclerosis in different animal models (Wang et al., 2011; Chen et al., 2016). To further investigate the molecular mechanism responsible for TMAO-induced endothelial dysfunction in aging, we measured expression of pro-inflammatory cytokines TNF-α, IL-1β, activity of eNOS, and levels of superoxide production in the aorta. Consistent with previous studies (Wang et al., 2007; Lesniewski et al., 2011; Hezel et al., 2016; Guers et al., 2017), we observed significant increase in expression of TNF-α and IL-1β, decrease in expression of total and phosphorylated eNOS, and enhanced superoxide production in the aorta of old rats. Moreover, we found that aging-induced changes in above molecular parameters were reversed by DMB treatment. These findings indicate that aging-associated increases in circulating TMAO levels account for vascular inflammation and oxidative stress, which lead to impaired eNOS-derived NO production and contribute to endothelial dysfunction during aging process (Figure 6). Interestingly, we did not find significant change in the ratio of phosphorylated eNOS to total eNOS in the aorta of old rats, which is consistent with previous report (Guers et al., 2017), suggesting that aging-associated changes are a consequence of an overall reduction in eNOS content rather than a reduction in eNOS phosphorylation. The precise mechanism by which TMAO decreases the expression of eNOS in the aorta of old rats is unclear. It is well-known that the formation of NO from eNOS in blood vessels is dependent on L-arginine, which can be degraded by enzyme arginase (Herrera et al., 2010). Upregulation of arginase has been demonstrated in the aorta of old animals and is associated with endothelial dysfunction, while inhibition of arginase prevents aging-associated endothelial dysfunction (Tanabe et al., 2003; Yepuri et al., 2012). We could not exclude the possibility that TMAO-induced inflammation upregulates arginase, leading to the decrease in eNOS expression in the aorta of old rats.

A limitation of the present study should be acknowledged. Because direct *in vivo* detection of NO is technically challenging, we did not directly measure NO generation from eNOS in the aorta. Further studies are necessary to directly measure NO production from eNOS in the aorta and determine whether elevated circulating TMAO levels are correlate with reduced NO production in aged animals.

In conclusion, the present study demonstrates that aging increases gut microbiota-dependent metabolite TMAO levels in the circulation, which lead to vascular inflammation and oxidative stress, contributing to endothelial dysfunction in aged rats. The findings from this study provide new insights into the mechanisms underlying endothelial dysfunction in aging. Interventions that reduce circulating TMAO levels may be a novel therapeutic strategy for prevention and treatment of aging-associated cardiovascular disease.

## Author contributions

Conceived and designed the experiment: TL and XL. Performed the experiments: TL, YC, and CG. Analyzed the data: TL, YC, and CG. Wrote the paper: TL.

### Conflict of interest statement

The authors declare that the research was conducted in the absence of any commercial or financial relationships that could be construed as a potential conflict of interest.
